# Sensing Techniques for Organochlorides through Intermolecular Interaction with Bicyclic Amidines

**DOI:** 10.3390/bios11110413

**Published:** 2021-10-23

**Authors:** Jong-Won Park, Lee-Woon Jang, Erik C. Jensen, Amanda Stockton, Jungkyu Kim

**Affiliations:** 1 Department of Mechanical Engineering, University of Utah, Salt Lake City, UT 84112, USA; jwonpark@gmail.com (J.-W.P.); jang2woon@gmail.com (L.-W.J.); 2 HJ Science and Technology, San Leandro, CA 94577, USA; erikjensen100@gmail.com; 3 Department of Chemistry, Georgia Institute of Technology, Atlanta, GA 30318, USA; astockto@gatech.edu

**Keywords:** micellar electrokinetic chromatography, capillary zone electrophoresis, organochloride detection, chlorinated hydrocarbons, halogen bonding, environmental pollution

## Abstract

Toxic organochloride molecules are widely used in industry for various purposes. With their high volatility, the direct detection of organochlorides in environmental samples is challenging. Here, a new organochloride detection mechanism using 1,5-diazabicyclo[4.3.0]non-5-ene (DBN) is introduced to simplify a sensing method with higher detection sensitivity. Three types of organochloride compounds-trichloroethylene (TCE), dichloromethane (DCM), and dichlorodiphenyltrichloroethane (DDT)—were targeted to understand DCM conjugation chemistry by using nuclear magnetic resonance (NMR) and liquid chromatography with a mass spectrometer (LC-MS). ^13^C-NMR spectra and LC-MS data indicated that DBN can be labeled on these organochloride compounds by chlorine–nitrogen interaction. Furthermore, to demonstrate the organochloride sensing capability, the labeling yield and limit of detection were determined by a colorimetric assay as well as micellar electrokinetic chromatography (MEKC). The interaction with DBN was most appreciable for TCE, among other organochlorides. TCE was detected at picomolar levels, which is two orders of magnitude lower than the maximum contaminant level set by the United States Environmental Protection Agency. MEKC, in conjunction with this DBN-labeling method, enables us to develop a field-deployable sensing platform for detecting toxic organochlorides with high sensitivity.

## 1. Introduction

Small organochlorine compounds, which are generally used as organic solvents, can cause neurological damage, endocrine disorders, and cancers [1,2,3,4]. Trichloroethylene (TCE), dichloromethane (DCM), and dichlorodiphenyltrichloroethane (DDT) are commonly used solvents in industry for various chemical processes. Due to the high stability and volatility of organochlorides, these remain intact in water, air, and soil for a long period of time. According to the United States Environmental Protection Agency (EPA) regulations, the maximum allowable contaminant levels of typical organochlorines in drinking water are as low as 5 ppb for TCE, 5 ppb for DCM, and 50 ppb for DDT [5,6,7]. When organochloride contamination occurs, contaminated sites should be cleaned through chemical, biological, and/or photolytic degradation processes [8,9,10,11,12,13,14,15,16]. Rapid assessment to determine the concentration and origin of organochloride compounds is crucial for effective remediation processes [17,18,19,20,21].

A variety of analytical methods have been used to detect and quantify organochlorides. GC/MS (gas chromatography/mass spectrometry) and liquid chromatography (LC) with a UV absorbance detector have been used as standard analytical methods for the analysis of organic pollutants in laboratories to obtain proper sensitivity [22,23]. However, these instruments are bulky and expensive and consume large amounts of organic solvents, which is far from being practical for field tests. A colorimetric assay can be an alternative method to overcome these drawbacks; however, this approach typically requires lengthy incubation times for chemical complexation with chromophores and shows poor sensitivity for the detection of small organochlorine compounds [24].

In this study, we developed a fluorescence-based assay for TCE, DCM, and DDT in an aqueous solution using the formation of organochloride-amidine complexes. We found that TCE molecules bind to 1,5-diazabicyclo[4.3.0]non-5-ene (DBN) through Cl···N interactions [25,26]. Typically, this cyclic amidine is used as a base in organic synthesis; however, it was used as a labeling agent in this study, along with amine-reactive Pacific Blue (PB) dye. The formation of organochloride-DBN complexes by Cl···N halogen bonding [27] was characterized and analyzed by 13C-NMR, LC/MS, and a colorimetric assay. With this labeling method, micellar electrokinetic chromatography (MEKC) [28] was performed using a portable microfluidic MEKC platform, and a picomolar level of LOD was achieved. This new labeling chemistry using a portable microfluidic MEKC platform demonstrates high sensitivity for the detection of organochlorides without bulky analytical instruments [29].

## 2. Materials and Methods

All organochlorides (TCE, DCM, and 4,4′-DDT), DBN, dimethyl sulfoxide (DMSO), sodium tetraborate, and sodium dodecyl sulfate (SDS) were obtained from Sigma-Aldrich (St. Louis, MO, USA). Pacific Blue succinimidyl ester (PB) was purchased from Thermo Fisher Scientific (Waltham, MA, USA). Four different concentrations (278 nM, 555 nM, 55.5 μM, and 5.55 mM) of organochlorides and 165 mM of DBN were prepared using DMSO. To prepare a mixture of organochloride and DBN, nine parts of each organochloride were mixed with one part of the DBN solution to obtain the final concentrations of 250 nM, 500 nM, 50 μM, and 5 mM for organochlorides and 16.5 mM for DBN. The viscosity and color of the mixture provide good indicators for estimating the preliminary formation of the organochloride-DBN complex.

To understand the formation of this complex, ^13^C-NMR spectra of organochlorides, DBN, and their mixture were measured by a JEOL ECS 400 MHz NMR spectrometer (Peabody, MA, USA). These spectra were obtained in a range of 10~180 ppm with 3.4 μs (30°) pulse and 2 relaxation delays. For measuring the spectrum of TCE only, 56.8 mM of TCE was prepared with DMSO (490 μL) and added to 120 μL of D_2_O to obtain a 45.6 mM TCE sample. For DBN, 165 mM of DBN was prepared with DMSO (490 μL) and added to 120 μL of D_2_O to obtain a 133 mM DBN sample. For the mixture of TCE and DBN, 245 μL of the 45.6 mM TCE and 245 μL of the 133 mM DBN for low TCE concentration and 245 μL of 182 mM TCE and 245 μL of the 133 mM DBN for high TCE concentration were added to 120 μL of D_2_O, respectively.

Liquid chromatography (LC)/electrospray ionization mass spectrometry (ESI-MS) studies were performed by a Dionex ultimate 3000 and a TSQ Vantage mass spectrometer (Waltham, MA, USA). The solvent for LC was a mixture of acetonitrile (2%) and formic acid (0.1%) in water. After 5 min, a mixture of 5 mM of each organochloride and 16.5 mM of DBN was separated by 150 mm × 75 μm Acclaim PepMap RSLC columns (Acclaim C18, Thermo Fisher Scientific Inc., Waltham, MA, USA) with a flow rate of 350 nL/min. The mass spectrometer was operated in a positive mode with an ESI voltage of 1500 V, and the scanned mass-to-charge ratio ranged from *m*/*z* 150 to *m*/*z* 800. Raw data from LC/ESI-MS were processed by an Xcalibur Qual Browser (Thermo Fisher Scientific, Waltham, MA, USA) [30].

As mentioned above, the color of the mixture changes with different concentrations of organochlorides. For such colorimetric detection of organochloride-DBN complexes, the concentration of each organochloride solution was adjusted with DI water to 250 nM, 500 nM, 50 μM, and 5 mM, respectively, and 16.5 mM of DBN solution was prepared with DMSO. After each organochloride and DBN were mixed, absorbance values at 450 nm were recorded at 0, 12.5, 25, 50, 125, and 250 h using a microplate reader, GENios (TECAN Group, Männedorf, Switzerland). 

Micellar electrokinetic chromatography (MEKC) was also performed using this labeling technique for the highly sensitive detection of each organochloride. A standard T-shaped microchip from Micralyne with a 50 µm width, 20 µm height, and 8 cm long separation channel was used [11]. 30 mM of sodium tetraborate at pH 9.2 with 60 mM of SDS was prepared for the MEKC running buffer. 125 nM, 250 nM, 500 nM, 50 μM, and 5 mM of each organochloride were prepared for the MEKC analysis. For the fluorescence labeling, amine-reactive PB was prepared at a concentration of 20 mM in DMSO. Five solutions of different organochloride concentrations (625 pM, 1.25 nM, 2.5 nM, 250 nM, and 25 μM) were prepared under a fixed concentration of PB (0.1 mM) and DBN (82.5 μM); the volume ratio used in this preparation was organochloride:PB:buffer:DBN = 1:1:200:1. After each mixture solution was left for 5 min at room temperature, the mixture was loaded into the capillary channel through the cross-injector with an applied voltage of 0, 0, −600, and 0 V to the anode electrode, sample, sample waste, and cathode electrode, respectively. After 60 s, the above voltage was changed to 0, −1500, −1500, and −8400 V, for separation of the mixture [28]. A custom-made fluorescence detection system using a 405 nm diode laser (Thorlab Inc., Newton, NJ, USA) [11] was used to obtain electropherograms of PB-labeled complexes from each sample. The electropherograms were also measured at 0, 2, 10, and 24 h, and peak areas of these electropherograms were calculated for obtaining conversion yields.

## 3. Results and Discussion

We observed a substantial change in the color of the organochloride solutions after mixing with DBN. Figure S1A shows the mixture of various concentrations of organochlorides and DBN in DMSO. The color intensities are proportional to the concentrations of organochlorides. In particular, TCE showed the most significant change among the organochlorides as compared with DCM and DDT. Furthermore, the viscosity of the mixture changes noticeably at high concentrations of TCE, as shown in Figure S1B. As the amount of TCE increases, the mixture turns into a gel-like solution, implying the formation of a cross-linked structure. 

**Figure 1:**
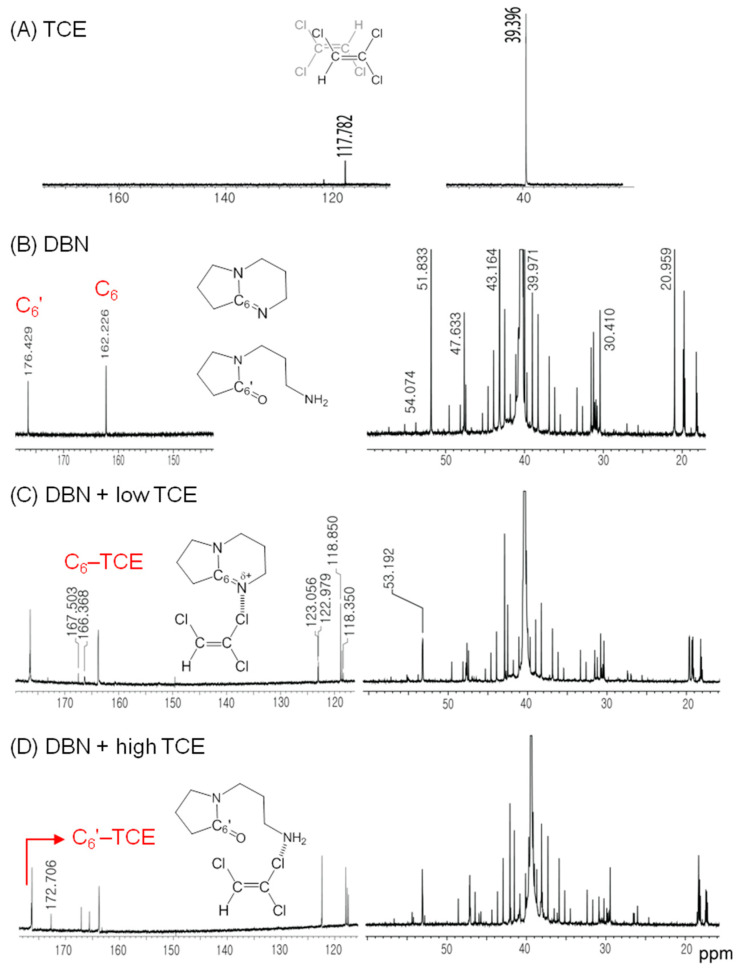
^13^C-NMR spectra of TCE, DBN, and their mixture. (**A**) TCE in DMSO/D_2_O. One major chemical shift at 117 ppm and the other minor shift at 122 ppm. (**B**) DBN in DMSO/D_2_O. 162 ppm: C_6_ carbon of DBN, 176 ppm: C_6_′ carbon of the hydrolyzed DBN. (**C**) The mixture of DBN and a low concentration of TCE. TCE interacts with DBN by Cl···N halogen bonding. C_6_-TCE: downfield shift of C_6_ carbon of DBN by the interaction with TCE. (**D**) The mixture of DBN and a high concentration of TCE. The amine group of the hydrolyzed DBN interacts with TCE, and the downfield shift of C_6_′ carbon is insignificant (labeled C_6_′-TCE). The peak intensity of TCE increases with the increase in TCE concentration. The (broad) peak around 39 ppm is DMSO.

To understand this chemical process, i.e., intermolecular interaction or molecular decomposition, we first investigated any possible change in the molecular structure of TCE and DBN using ^13^C-NMR spectroscopy. Figure 1 shows ^13^C-NMR spectra of TCE, DBN, and their mixtures. The mixtures with two molar ratios (1:2.9 and 4:2.9) of TCE to DBN were prepared for the NMR studies. TCE only in DMSO/D_2_O shows typical chemical shift (δ) at 117 ppm and 122 ppm (Figure 1A). The former is associated with the carbon bonded to the hydrogen atom. However, the δ values of DBN in DMSO/D_2_O display an indication of DBN decomposition [31]. The unexpected position of δ at 176 ppm [32], which is far downfield compared to the C_6_ carbon of pure DBN at 162 ppm (top structure in Figure 1B), is attributed to the hydrolyzed DBN [33,34], (3-Aminopropyl)-2-pyrrolidinone (bottom structure in Figure 1B). Water in the DMSO/D_2_O likely caused the hydrolysis of DBN [33], and hydrolysis is the only pathway for DBN decomposition in the experimental condition described above. The ^13^C-NMR indicated that the DBN and the hydrolyzed DBN do not interact with each other in the neutral condition since the chemical shifts represent the mere mixture of the two compounds. A detailed structure of DBN and NMR assignment can be found in the literature [31].

Figure 1C,D present ^13^C-NMR spectra of the mixture of DBN and two different TCE concentrations. Both spectra show similar chemical shifts. Compared to the NMR spectra from only TCE or DBN (Figure 1A,B), TCE in the mixture does not show a new peak; however, DBN in the mixture shows new peaks whereas other major peaks show the same δ values. Thus, we concluded that there exist intermolecular interactions between TCE and DBN without any noticeable chemical reactions [35,36]. The most plausible intermolecular interaction is a chloride–nitrogen interaction. When TCE interacts with DBN by Cl···N halogen bonding, the C_6_ carbon of the DBN becomes electron-deficient with the partially positive charge (δ+) on the nitrogen atom, and this is consistent with the downfield shift of δ = 162 ppm to δ = 167 ppm as shown in Figure 1C. Each δ of the TCE carbons at 117 ppm and 122 ppm splits into a doublet. In addition, the NMR peak in the interaction of TCE with the hydrolyzed DBN becomes distinct at a high concentration of TCE, which is located on the downfield side of δ = 176 ppm (Figure 1D). The presence of δ = 172 ppm may be due to the interaction of the oxygen atom of the hydrolyzed DBN with the hydrogen atom of the TCE since C6′ carbon should be electron-rich due to hydrogen bonding. Unlike aromaticity and polarity as the adsorption mechanism of TCE, for example, on biochars [37], Cl···N halogen bonding was identified as a more detailed type of interaction.

**Figure 2:**
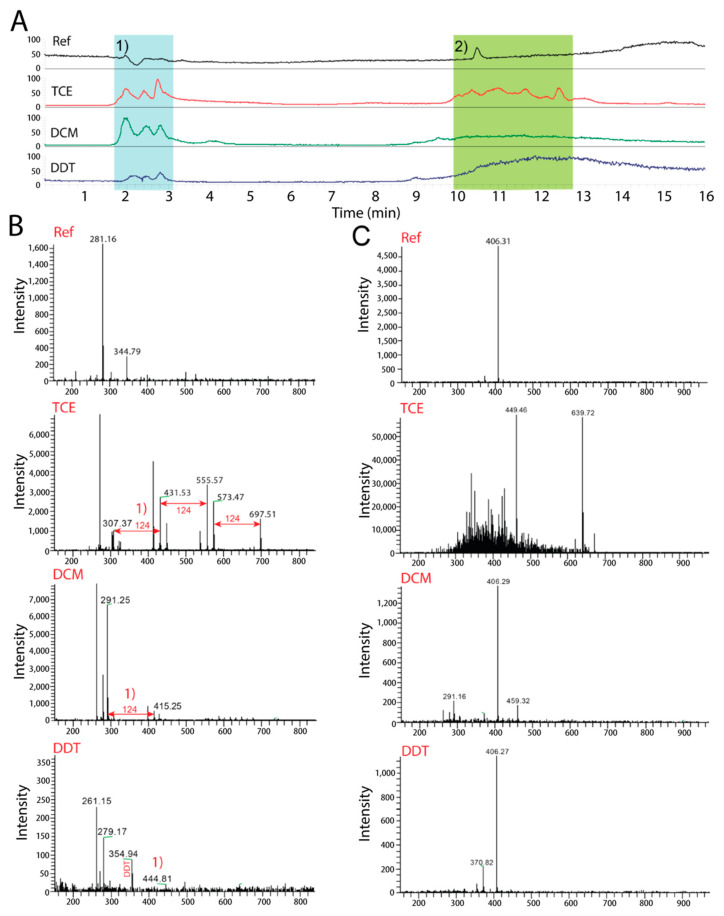
LC/MS data of the mixture of DBN and organochlorides. (**A**) LC chromatograms of the reference (DBN) and its mixture with organochlorides. (**B**) MS spectra from the short retention time (blue region) and (**C**) MS spectra from the long retention time (green region). The unit of the horizontal axis is *m*/*z*.

In addition to these ^13^C-NMR analyses, we analyzed the mixture using LC/MS to clarify the nature of this intermolecular interaction. Figure 2A shows LC chromatograms of the mixture of 5 mM of organochlorides and 16.5 mM of DBN. The background reference is DBN in a formic acid solution, and an additional peak is consistent with the partial hydrolysis of DBN. This acidic condition will produce more protonated forms of (hydrolyzed) DBN. The peaks at 2 min in Figure 2A are largely due to both pure DBN and hydrolyzed DBN, whereas the peaks at 10.5 min are mainly due to the hydrolyzed DBN, and this assignment is in good agreement with a mass analysis, as will be discussed later. Compared to DBN only, all the chromatograms of the organochloride-DBN mixtures presented two major changes. First, there are a series of three peaks in the short retention time indicated in region 1 of Figure 2A, and from the order of retention time, the peaks could be from DBN, hydrolyzed DBN, and their adduct, by estimating their molecular size: DBN (124) < hydrolyzed DBN (142) < hydrolyzed DBN-DBN adduct (266). Secondly, broadened peaks in the long retention time (region 2:10—13 min) were observed, possibly due to the interaction of the hydrolyzed DBN ammonium with the organochlorides. Hydrolyzed DBN molecules can have a positive charge on the protonated amine group, and this resulted in the appearance at the long retention time. In particular, both the carbonyl and amine groups of the hydrolyzed DBN interact with TCE (Figure 1D). This strong interaction of TCE to the (hydrolyzed) DBN molecules produced multiple peaks at the long retention time, such as TCE—(hydrolyzed) DBN + ion, TCE—(hydrolyzed) DBN-TCE, or TCE—(hydrolyzed) DBN-TCE—(hydrolyzed) DBN.

Mass spectra coupled with the LC chromatograms were obtained to gain more conclusive information on the organochloride-DBN complex. Figure 2B,C show the mass spectra recorded at the short and long retention times, respectively. The primary mass-to-charge ratio (*m*/*z*) of DBN, TCE, DCM, and DDT is *m*/*z* 124, *m*/*z* 134, *m*/*z* 84.9, and *m*/*z* 354.9, respectively [38]. A mass spectrum of DBN shows several major peaks at *m*/*z* 281.16, *m*/*z* 344.79, and *m*/*z* 406.31. We did not see molecular ions smaller than *m*/*z* 160, and this was due to the high concentration of the samples. Having excluded the possible formation of fragment adducts from ammonium (*m*/*z* 18) and acetamide (*m*/*z* 45) [39], we interpreted the *m*/*z* values as follows: 281 = DBN (124) + [hydrolyzed DBN] (142) + CH_3_ (15), 344—281 = 63 = DMSO (78)—CH_3_ (15), 406 = 2 × ([hydrolyzed DBN] + CH_3_COOH + H^+^) [33,40] = 2 × (142 + 61) in the acidic condition. The interpretation of the mass spectrum was made with the inclusion of the hydrolyzed DBN. Therefore, the mass analysis indicates that DBN exists in the form of the complexation between pure DBN and hydrolyzed DBN, i.e., (3-Aminopropyl)-2-pyrrolidinone.

**Scheme 1:**
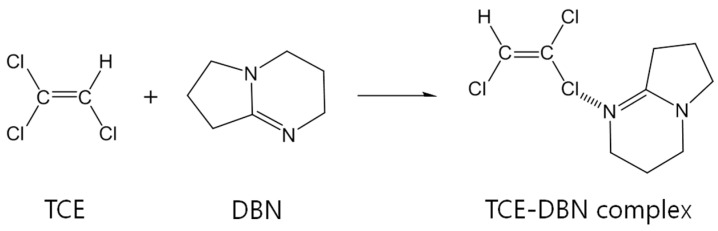
Complexation of TCE with DBN by Cl···N halogen bond.

The most remarkable result in the mass spectra of the organochloride-DBN mixtures is that the interval of *m*/*z* values is 124, which is not a fragment, but the mass of DBN. For the DBN + TCE mixture, the *m*/*z* value increases by 124 three consecutive times from *m*/*z* 307.37 to *m*/*z* 697.51 (the other repeated is *m*/*z* 142: the mass of the hydrolyzed DBN, Figure S2). The *m*/*z* 307.37 was interpreted as [TCE (134) + H^+^(1) + TCE (134)] + HCl (38). In the case of the DCM and DBN mixture, the peak interval between *m*/*z* 291.25 and *m*/*z* 415.25 is the same as TCE. Therefore, we hypothesized that one molecule of TCE with three chlorine atoms can bond with up to three DBN molecules, and one molecule of DCM with two chlorine atoms bonds mostly with one DBN molecule. This result implies that the interaction of DBN is the most significant for TCE. With the characteristic molecular ion of DDT observed at *m*/*z* 354.94, the lack of the *m*/*z* 124 interval in the DDT-DBN sample may indicate the weak interaction between DDT and DBN. Scheme 1 illustrates a probable mechanism of the intermolecular interaction of TCE with DBN through Cl···N halogen bonding. Note that the resonance form of DBN produces a partial positive charge on the nitrogen atom [31,32].

In Figure 2C, the sample of TCE + DBN shows numerous unresolved peaks at *m*/*z* 300~600, whereas the samples of DCM + DBN and DDT + DBN show a similar pattern with DBN only [41]. Thus, the interaction of the hydrolyzed DBN with TCE is much stronger than that of pure DBN with TCE. This interaction may play a role in the gel-like formation [42,43,44], and the hydrolyzed DBN does not interact strongly with DCM or DDT. In TCE, the difference between *m*/*z* 449 and *m*/*z* 640 is 191, and this can be assigned to DBN and its fragment (*m*/*z* 124 + *m*/*z* 67). Accordingly, *m*/*z* 449 was interpreted as *m*/*z* 124 + *m*/*z* 134 + Δ*m*/*z* 191, which is consistent with the existence of DBN-TCE complexes. From these mass spectrometry analyses, we were able to determine the possible molar ratio in the organochloride-DBN complex and to estimate the relative strength of the organochloride-DBN complex: TCE > DCM >> DDT. This strength of the interaction shows excellent agreement with the absorption and fluorescence study about complexation in the following discussions.

**Figure 3:**
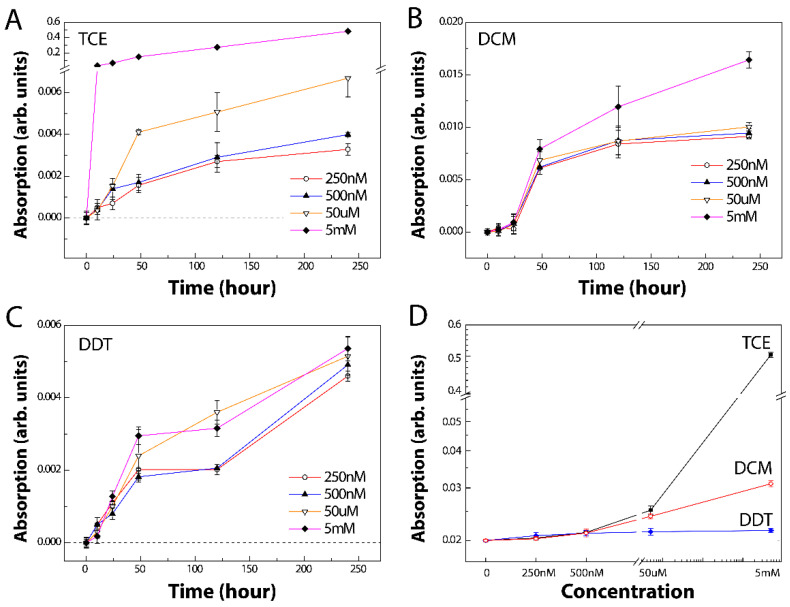
Limit of detection of three organochlorides measured at a wavelength of 450 nm in the presence of DBN. (**A**) TCE, (**B**) DCM, (**C**) DDT. Absorption was recorded for 240 h. (**D**) Absorption with various TCE, DCM, and DDT concentrations.

We monitored the absorption of the organochloride-DBN complexes at 450 nm upon the addition of DBN to obtain preliminary information on complexation yield and limit of detection (LOD). Figure 3 shows the absorption profile of each organochloride versus time in the presence of DBN. The absorption of TCE-DBN complexes increases remarkably by two orders of magnitude as the concentration of TCE increases. In the case of DCM and DDT, however, the increase in absorption is negligible. Therefore, the interaction between organochlorides and DBN is the most significant for TCE. All the absorption profiles indicate that most of the organochloride-DBN complexes are formed during the first 50 h. Based on the absorption versus organochloride concentrations shown in Figure 3D, the LOD was estimated to be approximately 200 nM. This colorimetric technique enables the simple and efficient quantification of the organochlorides, although it takes a long time to complete the detection procedure with relatively low sensitivity.

**Figure 4:**
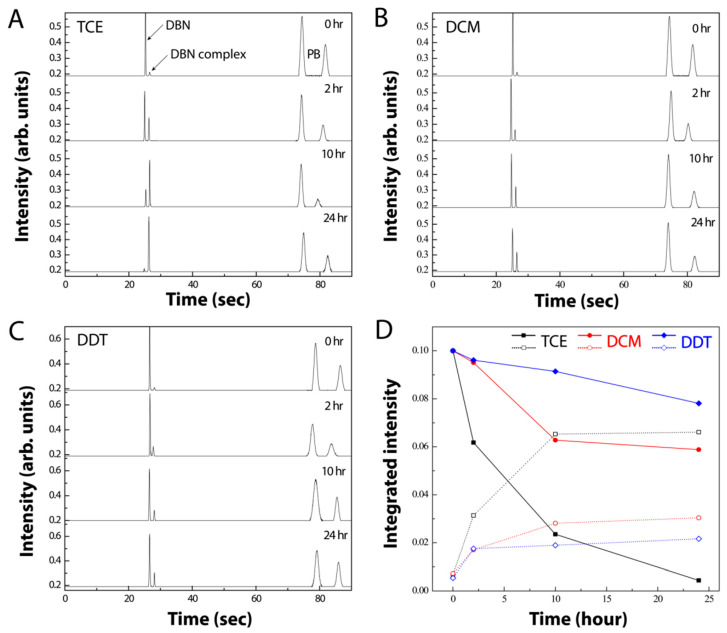
Electropherograms of the mixtures of DBN and organochlorides (**A**) TCE, (**B**) DCM, and (**C**) DDT, measured at different reaction times. From the control experiments, there are no obvious peaks that appeared from DBN + PB and TCE + PB. Peak intensities reflect the concentration of PB-coupled chemical species. (**D**) Conversion yield plots obtained from the area of DBN and organochloride-DBN complex peaks with reaction times. Solid lines with closed circles for DBN; dotted lines with open circles for organochloride-DBN complex.

To improve the overall sensing time and sensitivity, we analyzed the organochloride-DBN complex by further labeling it with PB fluorescence dye using a MEKC chromatography technique. The fluorescent PB is typically used as an indicator of the presence of amine molecules with the formation of an amide bond. However, in this study, the PB has proven to be labeled on the organochloride-DBN complex, likely by intermolecular interaction or possibly in micelles that may form when the concentration of DBN is high [44]. The mechanistic detail of the interaction is outside the scope of the MEKC analysis. The electropherograms of the MEKC analyses show that the fluorescence signal of the organochloride-DBN complex is separated from that of DBN due to the difference in molecular size and electrical charge [45]. This led us to explore the complex formation with various reaction times and organochloride concentrations to understand the reaction kinetics and the detection limit. Figure 4 shows the electropherograms from the mixtures of each organochloride (25 μM) and DBN (82.5 μM) at different mixing durations. All mixtures were incubated with PB for 5 min before the MEKC analyses. 

**Figure 5:**
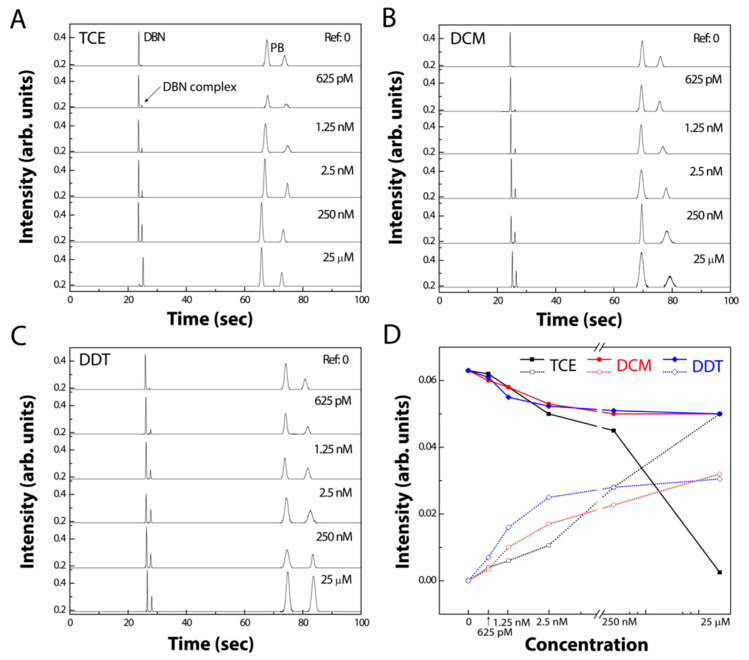
Electropherograms of the mixture of DBN and various concentrations (0~25 μM) of (**A**) TCE, (**B**) DCM, and (**C**) DDT. (**D**) A plot obtained from the peak area of DBN and organochloride-DBN complex with various concentrations. Solid lines with closed circles for DBN; dotted lines with open circles for organochloride-DBN complex.

To estimate the limit of detection (LOD) of the organochlorides in the MEKC analyses, 625 pM, 1.25 nM, 2.5 nM, 250 nM, and 25 μM of each organochloride was mixed with DBN and PB, and then incubated for 24 hrs before loading. As shown in Figure 5 (top electropherograms), all the reference samples without organochlorides show only the peaks from DBN and PB. Once organochlorides were added, a peak of the organochloride-DBN complex appeared. In Figure 5A, for the DBN and the TCE-DBN complex, as the TCE concentration increased from 625 pM to 25 μM, the intensity of the TCE-DBN complex increased. In particular, the TCE-DBN complex was completely dominant over the DBN at a DBN concentration of 25 μM, where the peak of the trimeric TCE_1_-DBN_3_ complex was dominantly observed. Figure 5C show the electropherograms of DCM and DDT, which exhibit a similar trend to that of TCE. However, due to the weak interaction of DCM and DDT with DBN, excess DBN remains such that the complexation has not been completed. To estimate the overall detection limit, the area of each peak was obtained and plotted in Figure 5D. Using the standard deviation and sensitivity [29,46], we calculated approximate LODs of 170 pM for TCE, 100 pM for DCM, and 70 pM for DDT (see SI for calculation). These picomolar LODs are similar to those from conventional mass spectrometry methods [47,48]. 

In Figure 4A–C, the intensity is proportional to the concentration of PB-conjugated chemical species. In the case of the sample with a 0 hr mixing duration, four peaks were observed corresponding to DBN, the organochloride-DBN complex, and two peaks from PB itself. As the mixing progressed, the peak intensity of DBN gradually decreased while the concentration of the organochloride-DBN complex increased. The concentrations of DBN and the TCE-DBN complex were almost completely switched after 24 h, as shown in Figure 4A, indicating a quantitative conversion to a trimeric TCE_1_-DBN_3_ complex. In the case of DCM and DDT, as shown in Figure 4B,C, however, such conversion was much less than that of TCE. Peak areas were plotted for each organochloride, as presented in Figure 4D. This plot shows the yield of the conversion from DBN to organochloride-DBN complexes. All the conversion profiles show typical first-order association kinetics. After 24 h, a conversion yield of 95% for TCE, 40% for DCM, and 20% for DDT was achieved, respectively. These results are consistent with the findings of the ^13^C-NMR, LC/MS, and absorption measurements. On account of the trimeric TCE_1_-DBN_3_ formed, the process of TCE-DBN complexation is expectedly the fastest among the three types of organochlorides.

## 4. Conclusions

In this study, we demonstrated that the Cl···N interaction can be used in the preparation of samples of organochlorides by adding DBN as a complexation agent. With TCE, DCM, and DDT mixed with DBN, various analytical analyses have been demonstrated for the occurrence of such halogen bonding. ^13^C-NMR and LC/MS data indicated the formation of organochloride-DBN complexes and the hydrolysis of DBN molecules in an aqueous solution. Interestingly, the complexation process from the interaction induced noticeable color and viscosity changes. By using this simple analytical method, a colorimetric assay has been performed with a limit of detection (LOD) of 200 nM for organochlorides after 240 h. To overcome the drawbacks of a long incubation period, a portable microfluidic MEKC analyzer was used with Pacific Blue fluorescence. This platform enables us to detect organochlorides with high sensitivity (LOD: 170 pM for TCE, 106 pM for DCM, and 72 pM for DDT). These LODs are two orders of magnitude lower than those suggested by the United States EPA. In future work, we will seek to further understand the role of the hydrolyzed DBN in the complexation and understand how PB interacts with DBN-organochloride complexes. Although DBN-based detection of organochlorides with MEKC analysis may not be readily applicable to all the chlorinated hydrocarbons due to structural selectivity, we expect that this complexation method will provide an efficient analytical route for the detection of environmental contaminants such as pesticides and chlorinated organic solvents. 

In addition, a portable microfluidic MEKC platform with a laser-induced fluorescence detector requires the use of fluorescence tags to label the organochloride-DBN complexes, and this limits the operating detection wavelengths to longer than 430 nm. To expand the wavelength range, the sensing platform can be altered to include a UV detector. This may enable the use of additional types of labeling agents such as hydroxycoumarin, which could interact with targets via Cl···O bonding.

## Data Availability

The data presented in this study are available on request from the corresponding author.

## Supplementary Materials

The following are available online at https://www.mdpi.com/article/10.3390/bios11110413/s1, Photograph of the mixtures of organochloride (TCE, DCM, and DDT) and DBN; mass spectrum of the mixture of TCE and DBN; calculation for LOD values.
