# Supplementary material for: Sensing Techniques for Organochlorides through Intermolecular Interaction with Bicyclic Amidines
Source: Biosensors (Basel). 2021 Oct 23;11(11):413. doi: 10.3390/bios11110413 (PMC8615940; doi:10.3390/bios11110413)
Supplement: Supplementary file 1 [file biosensors-11-00413-s001.zip › biosensors-1401338-supplementary.pdf]

biosensors

MDPI

Article

Sensing Techniques for Organochlorides through Intermolecular Interaction with Bicyclic Amidines

Jong-Won Park [1,†], Lee-Woon Jang [1,†], Erik C. Jensen [2], Amanda Stockton [3] and Jungkyu Kim [1,*]

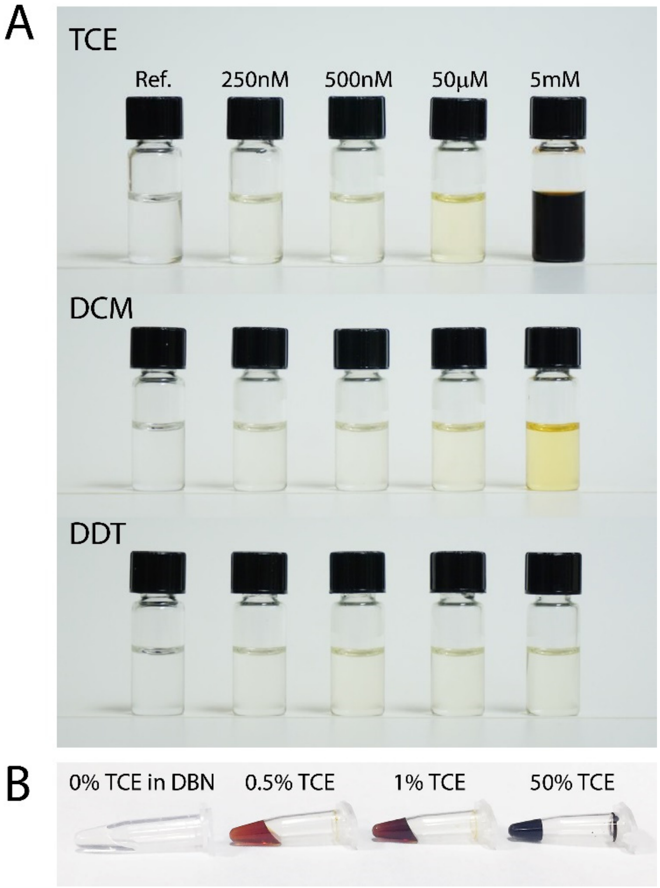

Figure S1. (A) Photograph of the mixtures of organochloride (TCE, DCM, and DDT) and DBN taken after 240 hours upon mixing. DBN concentration is 16.5 mM. (B) Photograph of TCE-DBN mixtures demonstrating a change of viscosity. The concentration of TCE at the 50% TCE sample is 90 mM in DMSO/H_2O (4/1). Note that viscosity increases as TCE concentration increases.

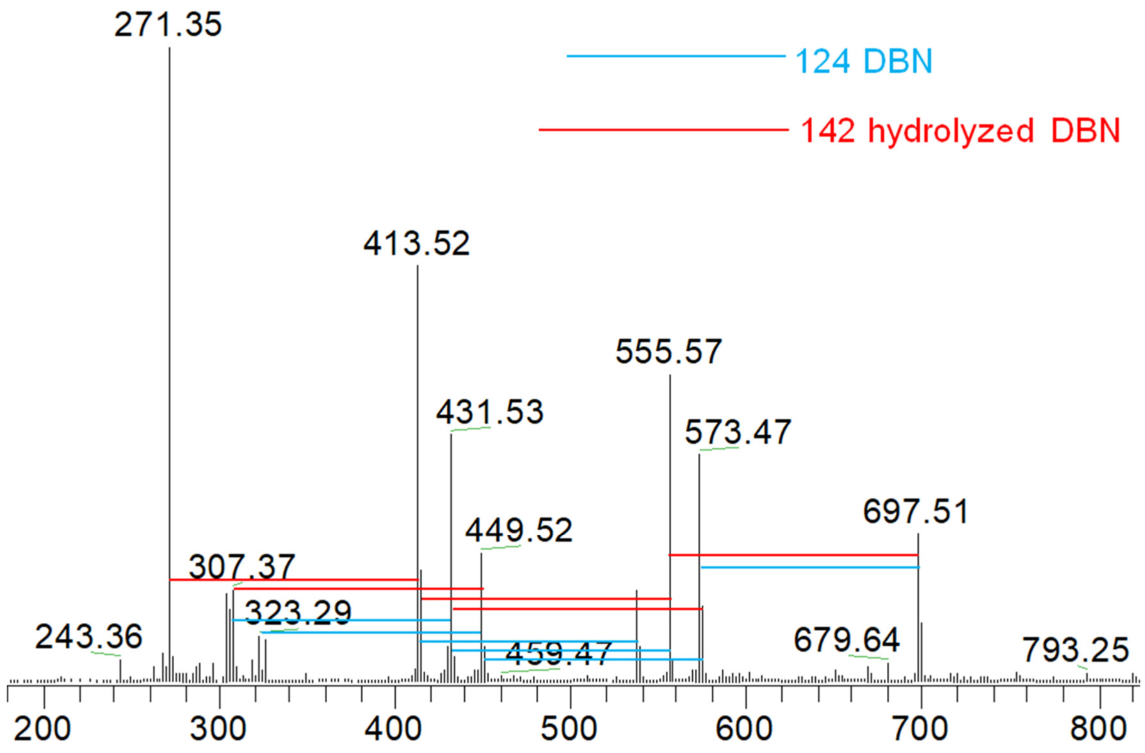

Figure S2. Mass spectrum of the mixture of TCE and DBN. Three consecutive intervals of m/z 124 (mass of DBN) and m/z 142 (mass of hydrolyzed DBN) are indicated.

The typical standard deviation (s) of the MEKC system (Figure S3 in Jang, L.-W., et al. *Lab Chip* **2016**, *16*, 3558) is 0.00022 at the blank (Ref) and 10 nM.

LOD[1,2]= 3.3s/sensitivity = 3.3s/slope

LOD (TCE) = 3.3×(0.00022)/(0.01055/2.5 nM)

= 0.172 nM

LOD (DCM) = 3.3×(0.00022)/(0.01706/2.5 nM)

= 0.106 nM

LOD (DDT) = 3.3×(0.00022)/(0.02500/2.5 nM)

= 0.072 nM

Reference

1. Kim, S.K.; Chang, H.; Zellers, E.T. Microfabricated Gas Chromatograph for the Selective Determination of Trichloroethylene Vapor at Sub-Parts-Per-Billion Concentrations in Complex Mixtures. *Anal. Chem.* **2011**, *83*, 7198–7206.
2. Belter, M.; Sajnóg, A.; Barałkiewicz, D. Over a century of detection and quantification capabilities in analytical chemistry—Historical overview and trends. *Talanta* **2014**, *129*, 606–616.
